# Account of Diseases in an Eastern District of London

**Published:** 1802-03

**Authors:** 


### ACCOUNT OF DISEASES IN AN EASTERN DISTRICT OF LONDON.

*From the 20th of January, to the 20th of February,* 1802.

| | |
|---|---|
| ACUTE DISEASES. | |
| Typhus Gravior | 1 |
| Typhus Mitior | 4 |
| Cynanche | 4 |
| Pneumonia | 6 |
| Peripneumonia Notha | 9 |
| Dyfenteria | 7 |
| Rheumatifmus Acutus | 5 |
| CHRONIC DISEASES. | |
| Tuffis | 27 |
| Tuffis cum Dyfpnœa | 14 |
| Dyfpnœa | 11 |
| Catarrhus | 3 |
| Hæmoptoe | 2 |
| Phthifis Pulmonalis | 5 |
| Pleurodyne | 4 |
| Hydrothorax | 2 |
| Anafarca | 7 |
| Palpitatio | 1 |
| Cephalalgia | 5 |
| Paralyfis | 2 |
| Enterodynia | 3 |
| Hæmorrhois | 2 |
| Vomitus | 3 |
| Dyfpepfia | 4 |
| Amenorrhœa | 12 |
| Dyfuria | 3 |
| Lumbago | 2 |
| Rheumatifmus Chronicus | 27 |
| PUERPERAL DISEASES. | |
| Menorrhagia Lochialis | 3 |
| Abfceffus Mammæ | 2 |
| Enurefis | 1 |
| INFANTILE DISEASES. | |
| Febris Mefenterica | 3 |
| Pertuffis | 7 |
| Diarrhœa | 3 |
| Herpes | 2 |
| Tinea | 1 |

Since the laft Report, the weather has undergone confiderable changes. For feveral days the temperature of the air was fo raifed, as to produce the feeling and appearance of a haftily approaching fpring; and during thofe few days, fome relief was experienced by patients labouring under the different difeafes of the thorax. Upon the return of cold and froft, however, the fymptoms attendant upon thefe complaints have again appeared, and in many cafes have been much aggravated. The difeafes referred to have generally prevailed amongft perfons far advanced in life; and to patients of this defcription, they have lately proved very fatal: But though they have been confidered as ranking rather in the fenile than the juvenile and infantile clafs of difeafes, they have lately appeared in feveral inftances amongft very young children, to whom they have been highly diftreffing, and, in too many inftances, fatal. Peripneumonic affections have, in fome of thefe cafes, conftituted the primary difeafe; but in others, they fucceeded a long-continued and fevere hooping-cough, by which the ftrength of the patient had been much reduced, and a conteft with the fupervening difeafe rendered more hazardous. In the former inftances, or where the peripneumonic fymptoms conftituted the primary difeafe, the ſymptoms were of the inflammatory kind, and yielded moſt readily to the antiphlogiſtic plan of treatment. The application of a leech or two to the breaſt, was in ſome inſtances followed by a mitigation of ſymptoms; the uſe of bliſters and different antimonial preparations, formed alſo an important part of the ſuccesſful treatment.

The advantage to be derived from expectoration is frequently loſt in the caſe of children, from the difficulty of prevailing upon them to part with the mucus which has been collected in the fauces; and on this account, the exhibition of emetic remedies, in a certain ſtage of the diſeaſe, becomes abſolutely neceſſary. In theſe caſes, a combination of ipecacuanha with the oxymel ſcillæ, given in ſuch doſes as to produce a nauſea of ſome continuance, previouſly to the full emetic effect, has been followed by a conſiderable mitigation of ſymptoms, and in ſome inſtances by a favorable termination of the diſeaſe. When a frequent repetition of this medicine is neceſſary to produce vomiting, it moſt commonly produces a ſalutary effect upon the bowels, and thus renders unneceſſary the exhibition of any other cathartic.

